# Preferences and Satisfaction Toward an Orthodontic Clinical App

**DOI:** 10.1055/s-0042-1760302

**Published:** 2023-02-22

**Authors:** Sasipa Thiradilok, Praeopailin Witayabusarakhum, Oranut Jearnsujitwimon, Somchai Manopatanakul

**Affiliations:** 1 Department of Advanced General Dentistry, Faculty of Dentistry, Mahidol University, Thailand; 2 Dentist, Computer Scientist, Bangkok, Thailand

**Keywords:** orthodontist, mobile applications, personal satisfaction

## Abstract

**Objectives** To promote the development of professional orthodontic apps and to grow app engagement, many contributing factors should first be scrutinized. The main purpose of this research was to assess whether gap analysis facilitates strategic app design.

**Materials and Methods** Gap analysis was first conducted to reveal users' preferences. Then, the OrthoAnalysis app was developed on an Android operating system using Java programming language. Finally, a self-administered survey was issued to 128 orthodontic specialists to assess their satisfaction toward usage of the app.

**Statistical Analysis** The content validity of the questionnaire was ascertained using an index of Item-Objective Congruence of more than 0.5. The reliability of the questionnaire was also analyzed with Cronbach's Alpha reliability coefficient (ɑ = 0.87).

**Results** Besides the most important factor, “content,” many issues were listed, and all were required to engage users. A strong and engaging app should show accurate, trustworthy, and practical clinical analysis that operates smoothly and fast with ease, along with a user-friendly, appealing, and trustworthy interface. In short, because of the preliminary gap analysis that was done to evaluate the potential app engagement power prior to app design, the result of the satisfaction assessment showed that nine traits including overall satisfaction were of high levels.

**Conclusions** Orthodontic specialists' preferences were assessed using gap analysis and an orthodontic app was designed and appraised. This article presents the orthodontic specialists' preferences and summarizes the process of achieving app satisfaction. Therefore, to create a clinical app with strong engagement power, a strategic initial plan using gap analysis can be recommended.

## Introduction

The increasingly used mobile applications or “apps” are a form of computer software programmed for mobile devices, smartphones, and tablets. Mobile devices equipped with dental apps would allow the performance of computerized functions to promote dental treatment and oral health whenever and wherever it is needed. Because of their easy access, efficiency, and versatility, apps developed for orthodontic purposes are desirable especially when meticulously designed. 1 2 3 4 However, apps specially designed for orthodontists are few in number. 5 6 There are even fewer orthodontic apps for clinical diagnosis, such as mixed dentition analysis. 7 Although the accuracy of orthodontic clinician apps has been investigated, 8 no research has yet been conducted to assess the practicality and engagement power of orthodontic apps. There is also a lack of research investigating the orthodontic specialists' expectations and perceptions toward such apps. Therefore, it was the aim of this study to assess the orthodontists' preferences and personal satisfaction toward the design and development of an orthodontic clinical app.

## Materials and Methods

This research was separated into two parts, the first being related to the development of an orthodontic app and the second was the assessment of the orthodontists' preferences and satisfaction about using the app by using a well-structured questionnaire.

### App Development

Prior to app design, gap analysis was done to identify the barriers and implementation factors relevant to the objective of this study to create a user-friendly app with engagement power. 9 Gap analysis was conducted by one orthodontist (SM) and one dentist who also was a computer programmer with a degree in computer science (PW). All available orthodontic clinician apps and related factors present at the time were assessed. 1 7 10 11 Gap analysis revealed that this app should initially be developed for the Android system (Trademark of Google, Mountain View, California, United States; www.google.com ). Consequently, the app was developed to compute pre-erupted tooth width prediction during mixed dentition stage and intermaxillary tooth width discrepancy which included widely accepted and used analyses, that is, Moyers analysis and user-customized international analysis. 12 13

### Questionnaire Design

The questionnaire consisted of two parts. The first part contained the general information of the study participants and their current mobile phone usage. The second part inquired about the preferences and satisfaction toward this orthodontic app.

In the first part, the demographic information of the participants included gender, age, and years of orthodontic experience. To survey their mobile phone usage information, seven questionnaire items were devised. These questions assessed the smartphone brand use (options provided were all phone brands available at the time of survey), app download frequency, preferred cost for downloading apps, app update frequency, hour(s) per day spent on the smartphone, future smartphone operating system of choice, and orthodontic application usage experience.

In the second part, the satisfaction assessment was verified in terms of content validity and reliability. Content validity was verified by two specialists (ST, SM) in the Faculty of Dentistry, Mahidol University, while reliability testing was performed on 10 participants. The evaluation of the reliability was done using Cronbach's ɑ coefficient tests. The questionnaire was then revised accordingly before being further used for this research.

Aspects of the satisfaction assessment using nine questionnaire items included app accessibility, utility, typeface, design, accuracy of operation, download span, ease of use, response time, and the overall satisfaction. The satisfaction scores were sorted into seven levels and were grouped into three categories, that is, high, moderate, and low using the evaluation criteria as suggested by Best and Kahn. 14

Finally, the last part consisted of three more questions. The first one surveyed the suitable price of this app. The second question was an indirect satisfaction assessment asking whether or not the participant would recommend this app to their friends. The final item was suggestions for the future development of this app.

This project was approved by the Institutional Review Board of the Faculty of Dentistry/ Faculty of Pharmacy, Mahidol University, Thailand (COA.No. MU-DT/PY-IRB 2014/023.2306). At the conference organized by the Thai Association of Orthodontists, participants were selected by the quota sampling method. Inclusion criteria were orthodontic specialists and student members of the Royal College of Dental Surgeons of Thailand.

Each participant's smartphone was then installed with the OrthoAnalysis app. If they did not own an Android smartphone, the app installed on a demo Android smartphone was used instead. Details of the research and instructions on using the app were explained. After the app trial by the participants, they were asked to fill in the questionnaire. Throughout the trial, any inquiries from the participants were explained directly by the researchers (SM, PW).

### Statistical Analysis

During the questionnaire development, the content validity of the questionnaire was ascertained using an index of Item-Objective Congruence (IOC). This index of IOC was more than 0.5, confirming complete agreement by all the experts. 15 The reliability of the questionnaire tested on 10 participants using Cronbach's Alpha showed high reliability with a value of *ɑ* = 0.87. 16

## Results

### App Development

**Fig. 1:**
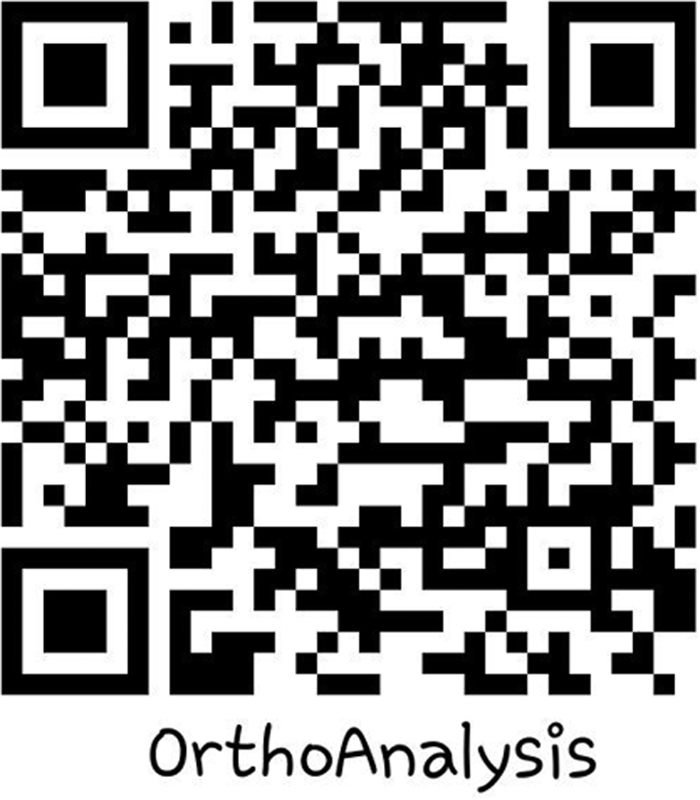
Quick Response code and logo of the OrthoAnalysis App.

**Fig. 2:**
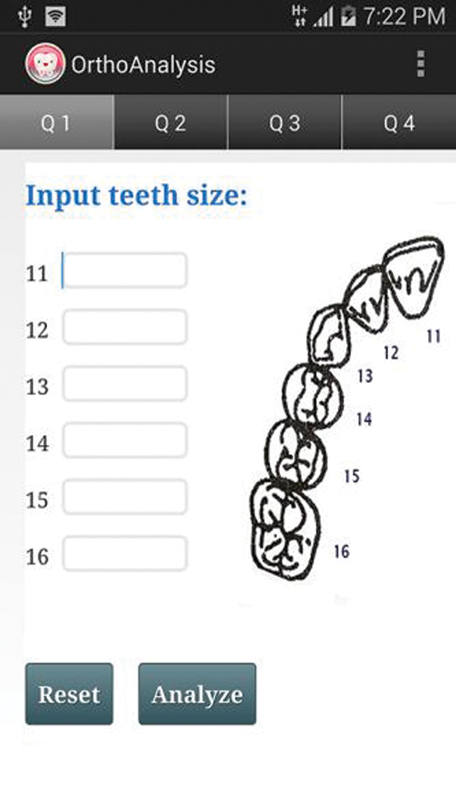
User input screens to enter tooth widths. Fédération Dentaire Internationale notations together with the tooth graphics were to ease the tooth width data input.

App development was performed based on the results of the gap analysis ( Table 1 ) which first revealed that the app should be initially developed on an Android system and should be easily accessible. Therefore, a Quick Response code for this Android app was generated for convenient app accessibility ( Fig. 1 ). Second, the operating system should be kept simple to obtain the shortest download and operating time. Because of this, Java programming language (Trademark of Oracle Corporation, Redwood City, California, United States; www.oracle.com ) was selected. Third, the app should offer a user-friendly input process, so this was achieved by only requiring the user to input the tooth width (in millimeters) into the provided space by using the smartphone's numeric keyboard and then simply selecting the preferred analysis ( Fig. 2 ); hence this input box should be clearly displayed. Finally, this app also provided equations for local and international use. Details on the app guide, display, interface, and clinical application were explained and published. 17

**Table 1:** Summary of the results of gap analysis for the factors leading to the best app design and most user-friendly app. The app implementation and barrier factors were also elicited and described

Best design and most user-friendly app	Implementing factors	Barrier factors	Apps which met these criteria
Easy access Fast download Fast opening time	Provide Quick Response (QR) code Select software that takes up the reasonable size of the memory of the mobile device, yet efficient	Inconvenience Slow down users' mobile device	iModelAnalysis I and II Model analysis Bolton Calc
“CONTENT” is top priority. Mandatory orthodontic mixed dentition analyses included Leads to excellence in clinical treatment	Various international formulas provided including both well-known equations and customized equations for accuracy Included analysis for further better clinical outcome	Insufficient analyses or analysis not suitable for patients from different racial groups Not include well-liked analyses	iModelAnalysis I and II
Easy tooth width data input Avoid confusion of different tooth numbering systems User-friendly and familiar interface App runs fast and smooth	Design input box for each tooth in the format of one quadrant per one screen Reinsert the contralateral tooth width automatically Clear overall screen display Appropriate display size of the fonts, numbers, and button and for every age group Text-to-speech function for reading the result out loud Clear, well-structured and understandable display on different brands of mobile devices Simple algorithm for the app to run the fastest	Confusion resulted from tooth numbering system while inserting tooth width Display layout not compatible with different brands of mobile devices	
Accurate, trustworthy, attractive, practical Stimulate engagement	Ready-to-use results Results in millimeters instead of proportions No further calculation or interpretation required Accurate calculation	Time-consuming process Meaningless or incorrect outcome	iModelAnalysis I and II
Appealing yet reliable design Display adaptable to all different phone screen dimensions	Clear responsive display with properly sized fonts and buttons Simple display design No harsh colors	Display cannot be read on small devices Boring design	OneCeph InterceptiveOrthodontics
Free of charge	Nonprofit app	High development and operating costs	OneCeph iModelAnalysis I and II OrthodonticCalculator
Available on both Android and iOS	Least rules/restrictions and no upload fee for Android	Many restrictions and require annual uploading fee as of iOS	

### General Information from Questionnaire

**Fig. 3:**
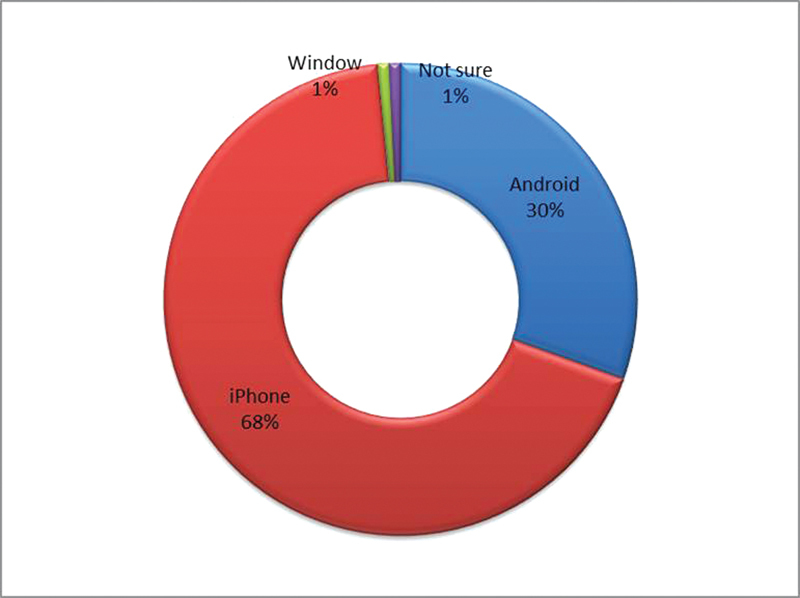
Smartphone operating system of choice: iPhone was the main preference (68%), followed by Android-based systems (30%).

Data were collected from 128 participants (excluding the nonresponse rate of 22%). The majority of the participants were female (62.5%) and were postgraduate students (45.3%). The age of the participants ranged from 25 to 62 years, with a mean age of 34.37 years (standard deviation = 9.03 years). Almost all participants were not familiar with any orthodontic applications (97.7%). Regarding a suitable price for the app, most participants would like it to be free of charge (55.5%). The preferred smartphone operating system was iPhone (68%), followed by Android (30%) ( Fig. 3 ). While most participants downloaded new apps less than once a month (43.8%), they updated their app mostly once or twice a month (25.0%). Most participants used the smartphone for 1 to 3 hours/day (45.3%). Moreover, almost all participants (98.4%) would recommend this app to others.

### Orthodontists' Satisfaction

**Fig. 4:**
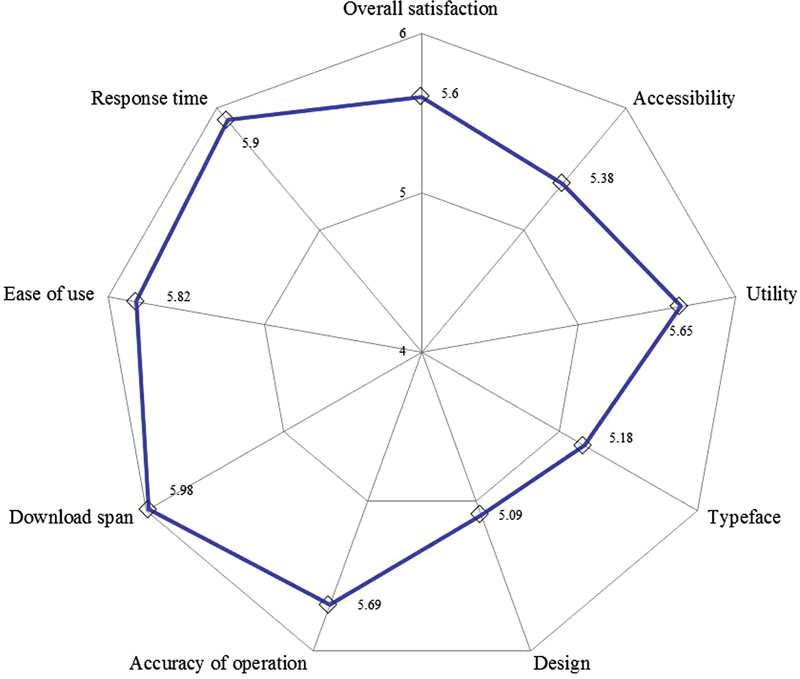
Radar chart showing the nine dimensions of the satisfaction assessment including overall satisfaction. All scores of greater than 5 (out of 7) were showed.

This study consisted of nine aspects of satisfaction including accessibility, utility, typeface, graphic design, accuracy, download span, ease of use, response time, and overall satisfaction. Scores of greater than 5 (out of 7) were shown in all aspects, indicating high satisfaction ( Fig. 4 ). Moreover, 33 participants proposed additional recommendations for this app, such as the incorporation of more analyses, for example, cephalometric analyses and also that the availability of this app should be extended to the iPhone operating system. Another suggestion was for some special features to be included such as voice command.

## Discussion

This research used gap analysis to direct the design and generation of the app of interest. Gap analysis has been developed and used in business management for a long time. It is also used to assess the performance of information technology or software applications to determine whether the objectives are being met. 9 Basically, the “gap” refers to the space between “where we are” and “where we want to be,” where the ultimate goal of this gap analysis would be to bridge all these gaps. To reach where we aspire the app to be, the app “content” is the main factor. To increase the engagement power, this app must be able to be downloaded and run speedily without delay. As orthodontists require the data input method to be simple, tooth display graphics were used to aid the input of tooth width data to avoid the confusion of different tooth numbering systems. The participants also indicated their preference for this app to be available on both Android and iOS. The most important requirements for this app design can be summarized as being kept simple, fast, and inexpensive, yet accurate, efficient, appealing, and trustworthy. Because of this, much attention and effort had been put into the app development stage to simplify the algorithm to achieve the fastest download and operating time.

The main strength of this study lies in the fact that, to our knowledge, it is the first study investigating the orthodontic specialists' expectations and perceptions toward orthodontic diagnostic apps. Further, it is the first study which apply gap analysis to facilitate the orthodontic app design. Finally, this tooth width analysis app was launched and ready for use, free of charge. 17

There are also limitations in this study. These include the fact that while this app comprehensively provides the tooth width prediction, it still does not support the tooth size arch size calculation. At this stage, this present app only provides tooth width analyses. In short, there are two main categories of tooth width analyses included in this app. First, tooth width prediction for mixed dentition analysis, Moyers' prediction was included. 12 Second to aid the orthodontic finishing, it provides Bolton analysis showing tooth width discrepancy of canine to canine and first molar to first molar. 18 To identify the problematic tooth causing misfit occlusion, Ho-Freer analysis was also equipped. 13 The application of this specialized tooth width analysis was documented. 19 Additionally, this app provides the Moyers prediction specifically for tooth width prediction for each part of Thailand. 20 21 22 23 Since Pancherz and Schäffer recommended the specific prediction for patients from exact part of Germany, 24 users who require the rigidly accurate tooth width prediction for their patients can create the most accurate prediction using “My equation” function. Additionally, for Ho-Freer analysis, this app also includes this special analysis specifically for Thai patients. 25 Finally, since this app lacks the arch size input, our future orthodontic apps will be developed to include a more comprehensive orthodontic mixed dentition analysis.

There are concern that orthodontic diagnosis apps are few in number. 5 6 Evaluating the apps for medical specialist, otorhinolaryngologists, Rak et al also posed their concern of medical specialist apps' lacking functionality and scientific integrity. 26 This study showed that content is the main factor in app design and that orthodontic content also follows the same trend as that of medical apps. Orthodontists undoubtedly require apps design, especially for those that could aid in diagnosis. Moreover, specialist apps be strategically designed to be fully functional. Simultaneously, they require more safety regulations. Further, content must be evidence-based. 27 28 Unquestionably, this app is no exception. 29 Orthodontists would also benefit from clinical apps that are able to display virtual treatment plans as well as apps that allow access to patient records, radiographs, and other data analysis. More provisions of strategized and comprehensive orthodontic apps for orthodontic diagnostic assistance are highly desirable.

## Conclusions

This research assessed orthodontists' preferences and satisfaction levels with this newly developed app, initially designed to run on Android smartphones. Assessment of the preferences was done by gap analysis, where “content” is the main engagement factor, followed by many others including fast, accurate, and responsive displays available for any device. The satisfaction assessment that was performed by using a systematically designed questionnaire revealed a high satisfaction rate among the majority of participants. Other suggestions that were received were the incorporation of additional content, development for iOS, and improvement in the user interface. The desire for strategy-driven orthodontic apps for the purposes of diagnosis assistance, and clinical use was also expressed.
